# Identification of stable senescence‐associated reference genes

**DOI:** 10.1111/acel.12911

**Published:** 2019-02-01

**Authors:** Alejandra Hernandez‐Segura, Richard Rubingh, Marco Demaria

**Affiliations:** ^1^ European Research Institute for the Biology of Ageing, University Medical Center Groningen University of Groningen Groningen The Netherlands

## Abstract

Cellular senescence is a state of permanent cell cycle arrest activated in response to damaging stimuli. Many hallmarks associated with senescent cells are measured by quantitative real‐time PCR (qPCR). As the selection of stable reference genes for interpretation of qPCR data is often overlooked, we performed a systematic review to understand normalization strategies entailed in experiments involving senescent cells. We found that, in violation of the Minimum Information for publication of qPCR Experiments (MIQE) guidelines, most reports used only one reference gene to normalize qPCR data, and that stability of the reference genes was either not tested or not reported. To identify new and more stable reference genes in senescent fibroblasts, we analyzed the Shapiro–Wilk normality test and the coefficient of variation per gene using in public RNAseq datasets. We then compared the new reference gene candidates with commonly used ones by using both RNAseq and qPCR data. Finally, we defined the best reference genes to be used universally or in a strain‐dependent manner. This study intends to raise awareness of the instability of classical reference genes in senescent cells and to serve as a first attempt to define guidelines for the selection of more reliable normalization methods.

Cellular senescence is a state of permanent cell cycle arrest activated by various damaging stimuli (Muñoz‐Espín & Serrano, 2014). Senescent cells develop several morphological and functional changes, from enlarged and misshaped cell body to secretion of various bioactive molecules—the senescence‐associated secretory phenotype (SASP). However, studies from many research groups, including ours, have highlighted that the senescence program is complex and heterogeneous (Chen, Ozanne, & Hales, 2005; Hernandez‐Segura, De Jong, Melov, Guryev, & Campisi, 2017; Wiley et al., 2017). Most, if not all, senescence‐associated markers are not senescence‐specific and often the classification of a cell as senescent is oversimplified. One of the most powerful techniques to monitor several senescence‐associated traits at is quantitative real‐time PCR (qPCR). qPCR is often used to measure the expression of senescence‐associated growth arrest markers, such as the Cyclin‐Dependent kinase inhibitors p16 and p21, of various SASP factors and of other effectors of morphological alterations, for example the down‐regulation of the nuclear lamina protein LMNB1 (Hernandez‐Segura, Brandenburg, & Demaria, 2018; Hernandez‐Segura, Nehme, & Demaria, 2018). qPCR is fast, accurate, relatively easy to perform, inexpensive and allows to measure multiple markers simultaneously. The interpretation of qPCR data heavily relies on the use of a normalization factor which is often calculated based on the expression of a reference gene—a gene whose levels remain unchanged among the different conditions analyzed (Dundas & Ling, 2012). The MIQE guidelines (Minimum Information for Publication of Quantitative Real‐Time PCR Experiments) also recommend to use at least two reference genes in every qPCR experiment (Bustin et al., 2009, 2013). In contrast, the common practice is to use a single housekeeping gene—a gene that covers an essential cellular function (Bustin et al., 2013; Chapman & Waldenström, 2015), despite housekeeping genes being not always stable (Eisenberg & Levanon, 2013). For example, GAPDH, a common housekeeping gene used for qPCR normalization, is unstable in many conditions and cell types (Eisenberg & Levanon, 2013; Kozera & Rapacz, 2013). Particularly in the senescence field, recent experiments of single‐cell qPCR—a variation of the qPCR that does not rely on the use of reference genes for normalization—reported changes in GAPDH expression in senescent vs. proliferating cells (Wiley et al., 2017).

In order to compile a list of the most common reference genes used to normalize qPCR in experiments involving senescent cells, we performed a systematic review of articles published in 2017 and 2018 which included senescent fibroblasts—arguably the most widely used cell type to model senescence in culture. Articles performing qPCR using microRNAs as a target were excluded since the normalization methods are still debated and are not comparable to other targets (Schwarzenbach, Da Silva, Calin, & Pantel, 2015). Our search (a description of it is provided in “Experimental Procedures”) yielded 105 results from which 48 were included after examination for availability of the required information and suitability according to stringent inclusion/exclusion criteria (Supporting Information Table S1). Only one article used RNA content to normalize the qPCR data, while all the others made use of reference genes. Despite the recommendation in the MIQE guidelines, the majority of articles (38/48 studies) used only one reference gene, while only two articles used two genes to normalize their qPCR data (Figure 1a). Remarkably, the remaining seven articles used different reference genes for different experiments within the same article or one reference gene for some experiments and two reference genes for some others. In these cases, the reasoning to use different normalization strategies in different experiments was not clear.

**Figure 1:**
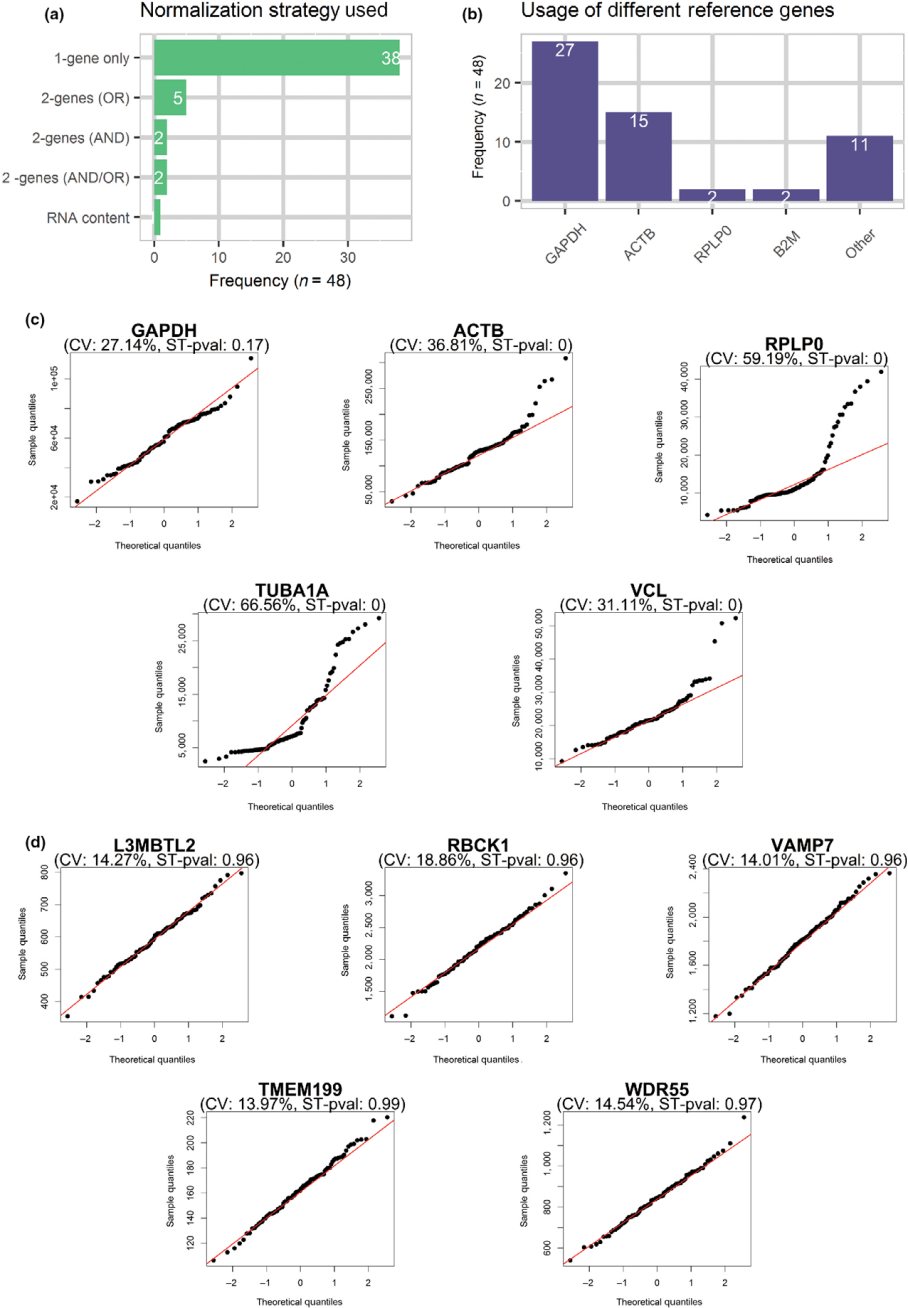
Reference genes for qPCR experiments including senescent cells are poorly stable. (a) Bar plot showing the method of choice to normalize qPCR data in experiments that include senescent fibroblasts. 1‐gene only = only one reference gene used to normalize data, 2‐genes (OR) = two different reference genes used one at a time for different experiments, 2‐genes (AND) = two reference genes used together to calculate a normalization factor, 2‐genes (AND/OR) = two reference genes used either one at a time or together in different experiments, RNA content = RNA content per sample used to normalize qPCR data (*n* = 48 articles). (b) Bar plot showing reference genes used in experiments that include senescent fibroblasts (*n* = 48 articles). The usage of a gene was counted regardless if it was used alone or in combination with another reference gene. (c) Quantile–Quantile plots for the expression of five reference genes commonly used to normalize qPCR data of senescent fibroblasts as evaluated by public RNAseq datasets. A total of 99 samples from ten different datasets were used to build the plots. The calculated CV and the p‐value for the Shapiro–Wilk normality test (ST‐pval). (d) Quantile–Quantile plots for the top five reference gene candidates picked having the highest ST‐pval and a CV lower than 20. RNAseq data for different fibroblast strains were used in combination with c and d

We also evaluated the frequency of specific reference genes. GAPDH was the most commonly used gene (27/48 studies) either alone or in combination with other reference genes. ACTB was the second most used reference gene (15/48 studies), followed by RPLP0 (2/48 studies) and B2M (2/48 times). Other genes (TBP, Rps29, GUSB, G6PD, Polr2a, HPRT, TFRC, SMARCA1, TUBA1A, and Rps13) were used in only one study each (Figure 1b). Of note, all the articles used a gene with a housekeeping function and none of them made clear whether the stability of the reference genes was tested beforehand.

A major issue is that several housekeeping functions, such as metabolism, cell structure, and protein synthesis, are altered in senescent cells (Hernandez‐Segura et al., 2017), and housekeeping genes might be differentially expressed in senescent samples (Eisenberg & Levanon, 2013; Zhang, Li, & Sun, 2015). To determine the stability of the most common reference genes used in experiments involving senescent cells, we analyzed ten public RNAseq datasets (Abdelmohsen et al., 2013; Alspach et al., 2014; Capell et al., 2016; Dikovskaya et al., 2015; Duarte et al., 2014; Hernandez‐Segura et al., 2017; Herranz et al., 2015; Marthandan et al., 2015, 2016; Rai et al., 2014). These datasets used different types of fibroblasts (foreskin fibroblasts: BJ, HFF, and HCA2 and lung fibroblasts: IMR90, MRC5, and WI38), and included proliferating, quiescent, and different types of pre‐ or fully senescent cells (induced by replicative senescence, oncogene‐induced senescence, and ionizing radiation‐induced senescence) (Supporting Information Table S2). We evaluated the stability of five commonly used reference genes: GAPDH, ACTB, and RPLP0, which were the top three reference genes identified in our systematic review analysis (Figure 1); TUBA1A, which our laboratory uses as reference; and VCL, often used as reference in protein expression experiments, namely Western blots. Following a similar strategy used by Yim et al. (2015), we evaluated the stability of each gene using these two criteria: (a) we assumed that the expression of reference genes should be stable in every sample independently of the condition. Therefore, the expression of a reference gene in all samples should follow a Gaussian distribution, which can be tested using a Shapiro–Wilk normality test; (b) the variation in expression among different samples, defined as coefficient of variation (CV), should be small for a reference gene. Following the indications provided by Yim et al. (2015), we considered that a stable and reliable reference gene should have a *p*‐value higher than 0.6 for the Shapiro–Wilk normality test and a CV lower than 20. Intriguingly, none of the common five reference genes passed the threshold (Figure 1c). We then expanded the analysis to every protein‐coding gene present in the pool of RNAseq datasets that we had collected. In this way, the reference gene candidates could be suitable for any of the cell strains and conditions contained in the datasets tested, avoiding the need to adapt several reference genes for routine studies that engage different senescence models. We identified 65 out of the 13,968 sequenced genes that met the criteria, and we selected the top five: L3MBTL2, RBCK1, TMEM199, VAMP7, and WDR55 (Supporting Information Table S3 and Figure 1d). The absolute expression levels of the five selected candidates were lower than common housekeeping genes, but high enough to be easily detected by qPCR (Supporting Information Figure S1). Moreover, there is no reason why genes that are expressed at a mid‐level would perform any worse than highly expressed genes in qPCR experiments (Eisenberg & Levanon, 2013).

Two analytical methods, namely geNorm and NormFinder, are commonly used for identification/validation of reference genes (Andersen, Jensen, & Ørntoft, 2004; Vandesompele et al., 2002). GeNorm uses the mean pairwise variation for a given reference gene candidate compared to the other candidates (*M*‐value) and excludes the least stable gene before repeating the analysis until only two (the most stable) genes are left. NormFinder uses a mathematical model of gene expression that measures the intra‐ and inter‐group variation of the candidate reference genes, giving a “stability value” as a result. In both cases, a lower *M*‐value and a lower stability value define the best reference gene. Both methods have pitfalls: geNorm is sensitive to gene co‐regulation, so two co‐regulated genes would maintain their pairwise variation despite not being stable. Indeed, some genes (mainly the commonly used ones) may be co‐regulated albeit evidence is not strong (Supporting Information Figure S2). NormFinder requires a bigger sample size per condition or treatment and, unlike geNorm, it does not provide a systematic way to choose the optimal number of reference genes required for a given experiment (De Spiegelaere et al., 2015). As both methods would be biased if used alone, we validated the stability of the candidate reference genes in qPCR experiments by combining them.

We generated 99 samples that included different strains of fibroblasts (BJ: 27 samples, HCA2: 27 samples, IMR90: 18 samples, and WI38: 27 samples) and different methods of senescence induction (doxorubicin, inhibition of different histone deacetylases, ionizing radiation, replicative senescence, and inhibition of DNA methylation; summarized in Supporting Information Table S4).

We measured the expression of ten reference gene candidates, the five commonly used (GAPDH, ACTB, RPLP0, TUBA1A, and VCL) and the novel five previously identified (L3MBTL2, RBCK1, VAMP7, TMEM199, and WDR55). We used geNorm and NormFinder to rank them according to their stability in each of the four cell types tested and in the combination of all of them together (Supporting Information Figure S3). Then, we built an overall ranking by averaging the information derived from the two methods (Figure 2b). For instance, if a gene scored 2 in geNorm and 4 in NormFinder, the overall rank would be 3. If two or more genes had the same overall ranking, the tie was solved by choosing the one with the lowest standard deviation for the overall ranking. This was done in order to avoid giving more weight to one of the reference gene selection methods. Overall, TMEM199 showed the highest stability and reliability among the tested reference genes (Figure 2b).

**Figure 2:**
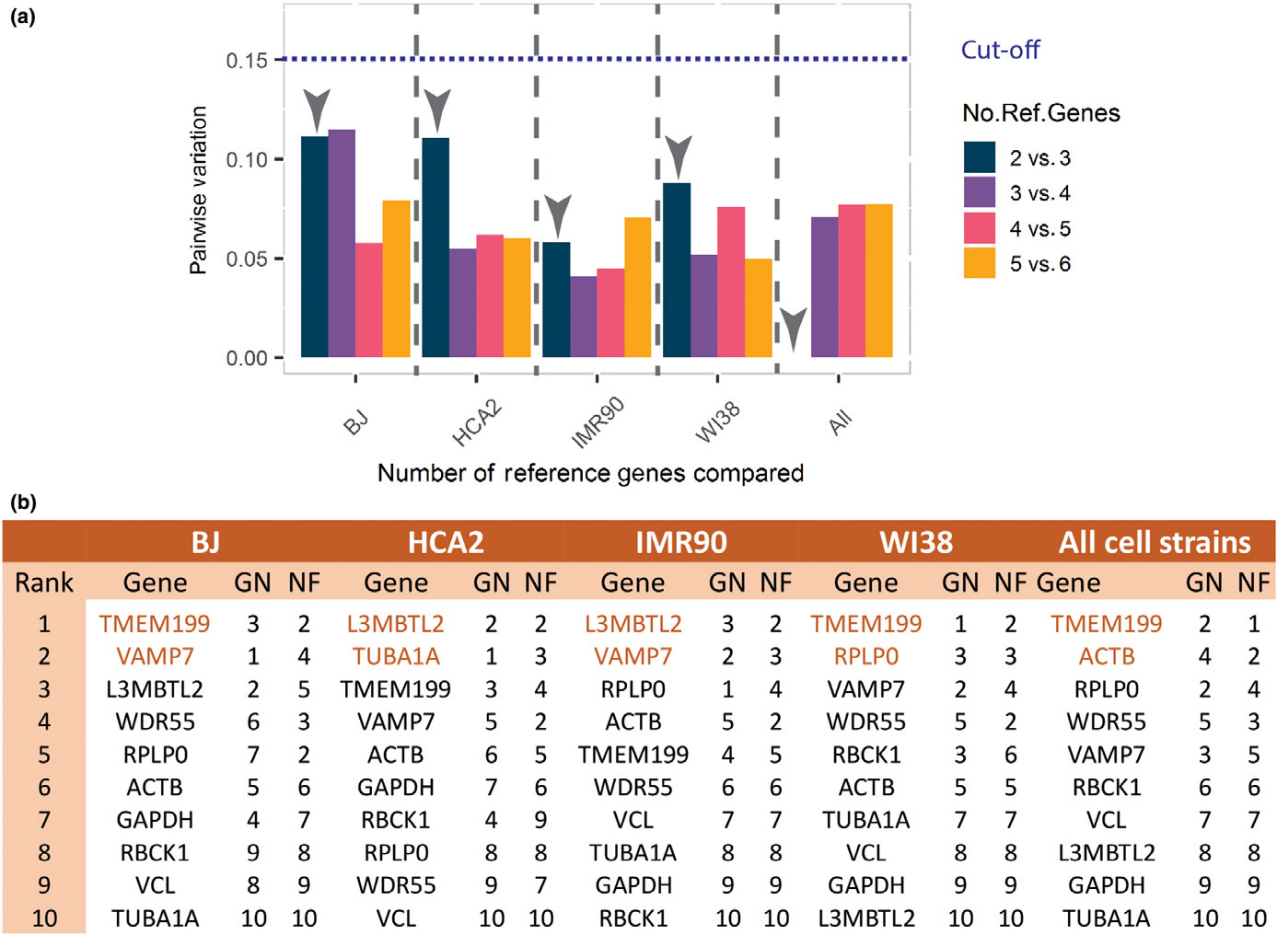
New candidates as reference genes to normalize qPCR data of senescent cells. The stability of the best reference gene was tested using qPCR data and the algorithms proposed by geNorm and NormFinder. (a) The normalization factor (geometric mean) using two, three, four, five, or six top reference genes were calculated for each cell type and for all cell types in combination (All). The performance of the different normalization factors was evaluated using geNorm. A difference in pairwise variation lower than 0.15 was used as a cutoff as recommended by Vandesompele et al. (2002). In all cases, two reference genes were sufficient for the calculation of the normalization factor. (b) Final ranking of the ten reference gene candidates tested by qPCR with both, geNorm (GN) and NormFinder (NF). Genes in orange mark the top two genes that were sufficient for the calculation of an adequate normalization factor

Finally, the MIQE guidelines suggest the use of at least two reference genes for every qPCR experiment and to test whether more than two are necessary. geNorm allows this evaluation by calculating the normalization factor (geometric mean of the expression of reference genes) combining the best two reference gene candidates and comparing it to the normalization factor using three, four, or more candidates. The pairwise variation of the different normalization factors is calculated, and a decision is taken on whether adding an extra gene would improve the analysis. In the original paper, it was proposed that if the use of an extra reference gene would decrease the pairwise variation more than 0.15 units, it would be necessary to include it in the normalization method. Following this protocol, we compared the performance of the normalization factor using two, three, four, five, or six reference genes (see Figure 2a). In all cases, the use of three genes did not significantly decreased the pairwise variation, so only the top two reference genes are necessary to normalize the qPCR data for the four cell types and conditions tested. This report and particularly the list shown in Figure 2b can be used as guidance for the selection of candidate genes in experiments involving senescent fibroblasts.

Some of the commonly used housekeeping genes that were not stable in the RNAseq data, ranked well in the qPCR data. These discrepancies might reflect the fact that the RNAseq analysis was used combining all the cell types together, so that stability in particular cell types is not tested. Moreover, the induction of senescence was not performed in the same way in both datasets. Another source of discrepancy might be the different transcript variants. Indeed, all the genes tested encode for multiple transcript variants which are all included in the RNAseq analysis. In contrast, the qPCR assays detect only a selection of those variants (see Supporting Information Table S5). In any case, our predicted candidates ranked generally better than the common reference genes.

With this report, we do not aim at criticizing experiments from other laboratories, but rather to raise awareness and encourage improvement. First, we cannot consider ourselves blameless because we used non‐tested and unstable genes as reference in previous studies, failing to critically address the problem of data normalization. Second, the conclusions stated in the articles used for the systematic review would probably hold, since in most cases different techniques were used to validate the findings. However, we believe that reproducibility of results would be improved if the description of the qPCR experiments would receive more attention.

We encourage choosing appropriate genes for every experiment tested, but the candidates suggested in Supporting Information Table S3 and Figure 2b set a starting point for genes to test. It is important that the field makes a shift toward better laboratory practices, particularly in times in which reproducibility of reports has been questioned (Baker & Dolgin, 2017; Begley & Ellis, 2012; eLife, 2017; Gutierrez, Mauriat, Pelloux, Bellini, & Van Wuytswinkel, 2008).

## CONFLICT OF INTEREST

None declared.

## AUTHOR CONTRIBUTIONS

Alejandra Hernandez‐Segura prepared most samples used, performed all the qPCR experiments, and the selection of reference genes both by RNAseq and qPCR. Richard Rubingh performed part of the selection of reference genes by RNAseq. Marco Demaria led and supervised the project. Marco Demaria and Alejandra Hernandez‐Segura formulated the idea and wrote the manuscript.
