# Supplementary material for: Identification of stable senescence‐associated reference genes
Source: Aging Cell. 2019 Feb 1;18(2):e12911. doi: 10.1111/acel.12911 (PMC6413663; doi:10.1111/acel.12911)
Supplement: Supplementary file 1 [file ACEL-18-e12911-s001.docx]

**SUPPORTING INFORMATION**

- **Experimental procedures**
- **Supplementary Tables (Table S1-S5)**
- **Supplementary Figures (Figure S1-S3)**

**EXPERIMENTAL PROCEDURES**

*Systematic review of reference genes used for studies including senescent cells*

We scouted for original scientific articles that used qPCR to measure gene expression in senescent versus proliferating/cancer/quiescent human fibroblasts. The search was done in PubMed (<https://www.ncbi.nlm.nih.gov/pubmed>) including only articles written in English between the beginning of 2017 until the moment of performing the systematic review (10/08/2018). The exact searching method used was: "senescence"[Title/Abstract] AND (("real-time polymerase chain reaction"[MeSH Terms] OR ("real-time"[All Fields] AND "polymerase"[All Fields] AND "chain"[All Fields] AND "reaction"[All Fields]) OR "real-time polymerase chain reaction"[All Fields] OR "qpcr"[All Fields]) OR ("gene expression"[MeSH Terms] OR ("gene"[All Fields] AND "expression"[All Fields]) OR "gene expression"[All Fields]) OR transcript[All Fields]) AND ("humans"[MeSH Terms] OR "humans"[All Fields] OR "human"[All Fields]) AND ("fibroblasts"[MeSH Terms] OR "fibroblasts"[All Fields]) AND (Journal Article[ptyp] AND ("2017/01/01"[PDAT] : "2018/08/10"[PDAT]) AND English[lang]). The search rendered 105 results that were further evaluated to ensure that they included bulk (no single-cell) qPCR experiments on human fibroblasts, qPCR of mRNAs or lncRNAs (no miRNAs) and that they included sufficient information about the normalization method of the qPCR data on the article or supplementary material. There was no restriction for the type of fibroblast used. Ten articles were excluded because we could not get access to the full-text. In total, we included 48 original articles. In each of them, we extracted the name of the gene(s) used to normalize the qPCR data or any other method used (if applicable).

*Acquisition, quality control and alignment of public RNAseq datasets*

Ten public datasets were collected from public repositories. The datasets can be found in the Gene Expression Omnibus under the following accession numbers: GSE77675, GSE56293G, GSE78138, GSE70668, GSE55949, GSE61130, GSE63577, GSE64553 and GSE53356 or in ArrayExpress database: E-MTAB-5403. Every dataset consisted on a set of senescent and proliferating fibroblasts (lung fibroblasts: WI-38, IMR90 or MRC-5 or foreskin fibroblasts: BJ,HFF or HCA-2), inducing senescence by different methods. A summary of the type of senescence, number of replicates and cell strain used in the generation of each dataset can be found on Table S2. A total of 99 samples were included in the analysis.

Raw data of the public datasets was downloaded as fastq files using the SRA Toolkit 2.6.2. Quality control of all samples was performed using the FastQC software v0.11.5 and the low quality reads (Average Quality: < 20) were discarded. End- trimming was performed when necessary by using the tool Trimmommatic 0.36. Samples were aligned to the GRCh38 genome using STAR-2.5.1b aligner and a count table was directly obtained with Star. Only genes annotated as protein-coding and showing more than 90 reads (while adding up the expression in all samples) were included in the analysis.

*Selection of reference genes based on RNAseq data*

Data was normalized to account for different sequencing depth in the different samples by calculating size factors using the DESeq2 software (Love, Huber, & Anders, 2014). The p-value of the Shapiro-Wilk normality test and the CV of each gene was calculated and used to evaluate the suitability of a gene to be used as a reference gene, as reported before (Yim et al., 2015). Genes that had a p-value higher than 0.6 and a CV lower than 20 were considered as reference gene candidates (see Table S3).

*Cell culture and senescence/quiescence induction*

Human foreskin fibroblasts HCA2 (male) were obtained from the laboratory of O. Pereira-Smith (University of Texas Health Science Center, San Antonio); human foreskin fibroblasts BJ were purchased from ATCC (Manassas, Virginia, USA, Cat: CRL-2522); human lung fibroblasts MRC-5 and human lung fibroblasts WI-38 were obtained from the laboratory of Judy Campisi (Buck Institute for Research on Aging, San Francisco). Each cell strain used was regularly monitored for mycoplasma contaminations (once/2 weeks). All cells were cultured in 5% oxygen and 37C for at least 3 Population Doublings (PD) prior to use and tested regularly for mycoplasma infection. Fibroblasts were cultured in DMEM (Thermo Fisher Scientific, Waltham, Massachusetts, USA, Cat: 31966-047) enriched with 10% fetal bovine serum (FBS, GE Healthcare Life Sciences, Chicago, Illinois, USA, Cat: 758092, Origin: South America, Batch: 41213-C05) and 1% penicillin/ streptomycin (Lonza, Switzerland, Cat: LO DE17-602E).

Quiescence was induced by culturing the cells for 48 hours in DMEM supplemented with 0.2% FBS.

Senescence was induced by different methods following standardized protocols (Hernandez-Segura, Brandenburg, et al., 2018). In brief, ionizing radiation-induced senescence, cells were subjected to a 10Gy dose of γ-radiation using a 137-Cesium source and medium was refreshed every 2 days. Cells were harvested at day 4, 10 or 20, as stated in Table S4. after irradiation for most of the experiments and validations.

For replicative senescence, cells were propagated in culture for ~3 months (re-cultured at 30%–40% density every time they reached 70%–80% confluence) until they slowed down their growth significantly (~PD 55 for BJ cells, with one population doubling every two weeks).

Doxorubicin (Tebu-bio, Netherlands, Cat: BIA-D1202-1) was used in a concentration of 250 nM in PBS for 24 hr. The medium was then replaced by normal DMEM supplemented with 10% FBS and refreshed every 2 days.

For epigenetic-induced senescence cells were treated with either 10 μM of 5-aza-2’-deoxycytidine (Sigma-Aldrich, St. Louis Missouri, USA, Cat: A3656), 1 μM of suberoylanilide hydroxamic acid (Sigma-Aldrich, St. Louis Missouri, USA, Cat. SML0061), 1 μM of RGFP966 or 1 μM of entinostat, all of them using DMSO as solvent. In every case, cells were treated for 3 days changing with fresh DMEM supplemented with 10% FBS medium + drug every 24 hours. Then they were cultured for 3 extra days with normal medium DMEM supplemented with 10% FBS.

Proliferating controls for each condition were generated stimulating cells with the corresponding vehicles and using cells of the same PD of the treated samples. In all cases (with the exception of the replicative senescence sample), cells were used between PD 35-40.

*Senescence-associated* β*–galactosidase assay*

Cells were plated in a 24-well plate, fixed in a mixture of gluteraldehyde and formaldehyde (2%/2%) for 10-15 min and stained over- night with an X-Gal solution as described in (Hernandez-Segura, Brandenburg, et al., 2018). Cells were counter-stained with a 1 mg/ml 40,6-diamidino-2-phenyl- indole (Sigma-Aldrich, St. Louis Missouri, USA, D9542) solution for 20 min. Images were acquired at 100X magnification, and the number of cells counted by the software ImageJ (<http://www.rsbweb.nih.gov/ij/>). The number of positive cells was counted manually in blind and the percentage of positive cells was calculated (data not shown).

*RNA extraction and cDNA synthesis*

Once the full treatment (if applicable) was performed, cells were collected in 350 μl of RNA Lysis buffer RLY (Bioline, UK, BIO-52079) and 3.5 μl of β-mercaptoethanol (Sigma-Aldrich, St. Louis Missouri, USA, M6250) using a cell scraper. Samples were frozen no more than 1 month at -80C before being processed. Total RNA was prepared using the Isolate II RNA Mini Kit (Bioline, UK, BIO-52073) following manufacturer’s instructions. 150–500 ng of RNA was reverse transcribed into cDNA using a kit (Applied Biosystems, Foster City, California, USA, Cat: 4368813) in a 20 μl reaction and incubating 10 min at 25C, 120min at 37C and 5 minutes at 85C. cDNA was diluted 6 times for a total volume of 120 μl before using it for qPCR.

*qPCR*

qPCR reactions were performed using the Universal Probe Library system (Roche, Switzerland, Cat: 04683633001) and a SENSIFast Probe kit (Bioline, UK, Cat: BIO-76001) in a 10 μl reaction from which 2.5μl corresponded to the diluted cDNA. Reaction was run in a Roche LightCycler 480 with the following program: 1) 7 min at 95C, 2) 40 cycles of 5 sec at 95c and 30sec at 60C and 3) 1 min at 37C. Each sample was run in duplicate.

All the primers were designed using the online software from Universal Probe Library Design Center (<https://lifescience.roche.com/en_nl/brands/universal-probe-library.html>). The primer sequences can be found in Table S5 and their respective experimental PCR efficiency and the r^2^ linearity of their standard curves in Table S6. Except for RBCK1 (87%), all the other primers had an efficiency of 95% or higher.

*qPCR data analysis*

The analysis to evaluate the suitability of a gene as a reference gene according to qPCR data was performed using the algorithms described for geNorm and NormFinder. In brief, the average Cq value from qPCR technical replicates was calculated. Measurements that were under the limit of detection were arbitrarily set to Cq=36 (limit of detection +1). The intra- and inter-group variation in the proliferating and senescent samples was calculated using an R function provided by NormFinder (<https://moma.dk/files/r.NormOldStab5.txt>) (Andersen et al., 2004).

For the geNorm analysis, the lowest Cq value for each gene was subtracted from all the other measurements. Then, the control stability measure M was calculated in R for each gene, followed by a stepwise exclusion of the least stable gene so that a list was constructed ranking the candidate reference genes according to their stability. The need of two or more reference genes was also calculated. The exact algorithms were described in (Vandesompele et al., 2002).

Co-regulation data used on Figure S2 was downloaded from <https://string-db.org> (version 10.5).

*Software used for analysis and plots*

All plots were made either in Excel 2016 or in R version 3.5.1 using the packages “ggplot2” and “DESeq2”.

**SUPPLEMENTARY TABLES**

- **Table S1: Evaluation of articles for inclusion/exclusion in the Systematic Review for Reference Genes used in the Senescence Field**

| **No.** | **Reference (pubmed)** | **Title** | **Authors** | **Inc.** | **Comments** |
| --- | --- | --- | --- | --- | --- |
| 1 | J Steroid Biochem Mol Biol. 2018 Sep;182:21-26. doi: 10.1016/j.jsbmb.2018.04.007. Epub 2018 Apr 17 | HMGA1a induces alternative splicing of estrogen receptor alpha in MCF-7 human breast cancer cells | Ohe K, Miyajima S, Abe I, Tanaka T, Hamaguchi Y, Harada Y, Horita Y, Beppu Y, Ito F, Yamasaki T, Terai H, Mori M, Murata Y, Tanabe M, Ashida K, Kobayashi K, Enjoji M, Yanase T, Harada N, Utsumi T, Mayeda A | No | No qPCR |
| 2 | (Al-Khalaf & Aboussekhra, 2018) | p16 Controls p53 Protein Expression Through miR-dependent Destabilization of MDM2 | Al-Khalaf HH, Aboussekhra A | Yes |  |
| 3 | (Macedo et al., 2018) | FoxM1 repression during human aging leads to mitotic decline and aneuploidy-driven full senescence | Macedo JC, Vaz S, Bakker B, Ribeiro R, Bakker PL, Escandell JM, Ferreira MG, Medema R, Foijer F, Logarinho E | Yes |  |
| 4 | (Hiebert et al., 2018) | Nrf2-Mediated Fibroblast Reprogramming Drives Cellular Senescence by Targeting the Matrisome | Hiebert P, Wietecha MS, Cangkrama M, Haertel E, Mavrogonatou E, Stumpe M, Steenbock H, Grossi S, Beer HD, Angel P, Brinckmann J, Kletsas D, Dengjel J, Werner S | Yes |  |
| 5 | Stem Cells Dev. 2018 Jul 1;27(13):922-934. doi: 10.1089/scd.2017.0275 | Human Mesenchymal Stromal Cell Sheets Induce Macrophages Predominantly to an Anti-Inflammatory Phenotype | Sukho P, Hesselink JW, Kops N, Kirpensteijn J, Verseijden F, Bastiaansen-Jenniskens YM | No | Different cell type used (macrophages) |
| 6 | (Nacarelli, Azar, Altinok, Orynbayeva, & Sell, 2018) | Rapamycin increases oxidative metabolism and enhances metabolic flexibility in human cardiac fibroblasts | Nacarelli T, Azar A, Altinok O, Orynbayeva Z, Sell C | Yes |  |
| 7 |  | The ING1a model of rapid cell senescence | Bertschmann J, Thalappilly S, Riabowol K | No | No qPCR |
| 8 | (Oh, Lee, Kim, Rhee, & Park, 2018) | Exosomes Derived from Human Induced Pluripotent Stem Cells Ameliorate the Aging of Skin Fibroblasts | Oh M, Lee J, Kim YJ, Rhee WJ, Park JH | Yes |  |
| 9 | Stem Cell Reports. 2018 May 8;10(5):1453-1463. doi: 10.1016/j.stemcr.2018.04.001 | Loss of MECP2 Leads to Activation of P53 and Neuronal Senescence | Ohashi M, Korsakova E, Allen D, Lee P, Fu K, Vargas BS, Cinkornpumin J, Salas C, Park JC, Germanguz I, Langerman J, Chronis C, Kuoy E, Tran S, Xiao X, Pellegrini M, Plath K, Lowry WE | No | Different cell type used (neurons derived from iPSC) |
| 10 | (von Muhlinen et al., 2018) | p53 isoforms regulate premature aging in human cells | von Muhlinen N, Horikawa I, Alam F, Isogaya K1, Lissa D, Vojtesek B, Lane DP, Harris CC | Yes |  |
| 11 | (Li, Li, Zhang, Yan, & Wang, 2018) | LncRNA RP11-670E13.6 Regulates Cell Cycle Progression in UVB Damaged Human Dermal Fibroblasts | Li M, Li L1, Zhang X, Yan Y, Wang B | Yes |  |
| 12 | (Fang et al., 2018) | Metformin alleviates human cellular aging by upregulating the endoplasmic reticulum glutathione peroxidase 7 | Fang J, Yang J, Wu X, Zhang G, Li T, Wang X, Zhang H, Wang CC, Liu GH, Wang L | Yes |  |
| 13 | Ann N Y Acad Sci. 2018 Apr;1418(1):95-105. doi: 10.1111/nyas.13566. Epub 2018 Jan 28 | Distinct patterns of gene expression in human cardiac fibroblasts exposed to rapamycin treatment or methionine restriction | Azar A, Lawrence I, Jofre S, Mell J, Sell C | No | No qPCR |
| 14 | (Mahemuti et al., 2018) | Bisphenol A induces DSB-ATM-p53 signaling leading to cell cycle arrest, senescence, autophagy, stress response, and estrogen release in human fetal lung fibroblasts | Mahemuti L, Chen Q, Coughlan MC, Qiao C, Chepelev NL, Florian M, Dong D, Woodworth RG, Yan J, Cao XL, Scoggan KA, Jin X, Willmore WG | Yes |  |
| 15 | (Qin, Zhang, & Zhang, 2018) | GSK126 (EZH2 inhibitor) interferes with ultraviolet A radiation-induced photoaging of human skin fibroblast cells | Qin H, Zhang G, Zhang L | Yes |  |
| 16 | (Lewinska et al., 2018) | Reduced levels of methyltransferase DNMT2 sensitize human fibroblasts to oxidative stress and DNA damage that is accompanied by changes in proliferation-related miRNA expression | Lewinska A, Adamczyk-Grochala J, Kwasniewicz E, Deregowska A, Semik E, Zabek T, Wnuk M | Yes |  |
| 17 | (Mrazkova et al., 2018) | Induction, regulation and roles of neural adhesion molecule L1CAM in cellular senescence | Mrazkova B, Dzijak R, Imrichova T, Kyjacova L, Barath P, Dzubak P, Holub D, Hajduch M, Nahacka Z, Andera L, Holicek P, Vasicova P, Sapega O, Bartek J, Hodny Z | Yes |  |
| 18 | Int J Mol Sci. 2018 Mar 27;19(4). pii: E1001. doi: 10.3390/ijms19041001 | Development and Characterisation of a Human Chronic Skin Wound Cell Line-Towards an Alternative for Animal Experimentation | Caley M, Wall IB, Peake M, Kipling D, Giles P, Thomas DW, Stephens P | No | Not about senescence |
| 19 | Sci Rep. 2018 Mar 20;8(1):4903. doi: 10.1038/s41598-018-23309-2 | The tobacco carcinogen NNK drives accumulation of DNMT1 at the GR promoter thereby reducing GR expression in untransformed lung fibroblasts | Taylor KM, Wheeler R, Singh N, Vosloo D, Ray DW, Sommer P | No | Not about senescence |
| 20 | Cancer Rep Rev. 2018 Mar;2(2). doi: 10.15761/CRR.1000145. Epub 2018 Jan 15 | Aberrant expression of p16INK4a in human cancers - a new biomarker? | Inoue K, Fry EA | No | Review |
| 21 | Oxid Med Cell Longev. 2018 Feb 13;2018:4814696. doi: 10.1155/2018/4814696. eCollection 2018 | Role of miR-200c in Myogenic Differentiation Impairment via p66Shc: Implication in Skeletal Muscle Regeneration of Dystrophic mdx Mice | D'Agostino M, Torcinaro A, Madaro L, Marchetti L, Sileno S, Beji S, Salis C, Proietti D, Imeneo G, C Capogrossi M, De Santa F, Magenta A | No | Not about senescence |
| 22 | (Piscitello et al., 2018) | AKT overactivation can suppress DNA repair via p70S6 kinase-dependent downregulation of MRE11 | Piscitello D, Varshney D, Lilla S, Vizioli MG, Reid C, Gorbunova V, Seluanov A, Gillespie DA, Adams PD | Yes |  |
| 23 | (E. K. Kim, Moon, Kim, Zhang, & Kim, 2018) | CXCL1 induces senescence of cancer-associated fibroblasts via autocrine loops in oral squamous cell carcinoma | Kim EK, Moon S, Kim DK, Zhang X, Kim J | Yes |  |
| 24 | Int J Biol Sci. 2018 Jan 14;14(2):165-177. doi: 10.7150/ijbs.23477. eCollection 2018 | Loss of p21 promoted tumorigenesis in the background of telomere dysfunctions induced by TRF2 and Wrn deficiency | Si X, Shao C, Li J, Jia S, Tang W, Zhang J, Yang J, Wu X, Luo Y | No | No qPCR data and used different cell type and organism (mouse embryonic fibroblasts) |
| 25 | Am J Chin Med. 2018;46(4):853-873. doi: 10.1142/S0192415X18500453. Epub 2018 May 8 | Restoring Effects of Natural Anti-Oxidant Quercetin on Cellular Senescent Human Dermal Fibroblasts | Sohn EJ, Kim JM, Kang SH, Kwon J, An HJ, Sung JS, Cho KA, Jang IS, Choi JS | No | No access |
| 26 | Proc Natl Acad Sci U S A. 2017 Dec 19;114(51):E10972-E10980. doi: 10.1073/pnas.1711613114. Epub 2017 Dec 1 | DNA replication timing alterations identify common markers between distinct progeroid diseases | Rivera-Mulia JC, Desprat R, Trevilla-Garcia C, Cornacchia D, Schwerer H, Sasaki T, Sima J, Fells T, Studer L, Lemaitre JM, Gilbert DM | No | No qPCR in senescent fibroblasts |
| 27 | (Saison-Ridinger et al., 2017) | Reprogramming pancreatic stellate cells via p53 activation: A putative target for pancreatic cancer therapy | Saison-Ridinger M, DelGiorno KE, Zhang T, Kraus A, French R, Jaquish D, Tsui C, Erikson G, Spike BT, Shokhirev MN, Liddle C, Yu RT, Downes M, Evans RM, Saghatelian A, Lowy AM, Wahl GM | Yes |  |
| 28 | Dig Dis Sci. 2017 Dec;62(12):3402-3414. doi: 10.1007/s10620-017-4794-5. Epub 2017 Oct 20 | Androgen Signaling in Esophageal Adenocarcinoma Cell Lines In Vitro | Palethorpe HM, Drew PA, Smith E | No | No qPCR insenescent fibroblasts |
| 29 | Genes Cells. 2017 Dec;22(12):982-992. doi: 10.1111/gtc.12542. Epub 2017 Nov 27 | Nicotinamide phosphoribosyltransferase delays cellular senescence by upregulating SIRT1 activity and antioxidant gene expression in mouse cells | Khaidizar FD, Nakahata Y, Kume A, Sumizawa K, Kohno K, Matsui T, Bessho Y | No | Used different cell type and organism (Mouse Embryonic Fibroblasts) |
| 30 | Mol Med Rep. 2017 Dec;16(6):9763-9769. doi: 10.3892/mmr.2017.7804. Epub 2017 Oct 17 | Protective effects of rosmarinic acid against hydrogen peroxide‑induced cellular senescence and the inflammatory response in normal human dermal fibroblasts | Hahn HJ, Kim KB, An IS, Ahn KJ, Han HJ | No | No access |
| 31 | Stem Cell Res Ther. 2017 Nov 15;8(1):263. doi: 10.1186/s13287-017-0711-2 | Thioredoxin mitigates radiation-induced hematopoietic stem cell injury in mice | Sundaramoorthy P, Wang Q, Zheng Z, Jiao Y, Chen BJ, Doan PL, Chao NJ, Kang | No | No qPCR |
| 32 | (Xiao et al., 2017) | Ozone oil promotes wound healing by increasing the migration of fibroblasts via PI3K/Akt/mTOR signaling pathway | Xiao W, Tang H, Wu M, Liao Y, Li K, Li L, Xu X | Yes |  |
| 33 | (Delestre et al., 2017) | Senescence is a Spi1-induced anti-proliferative mechanism in primary hematopoietic cells | Delestré L, Cui H, Esposito M, Quiveron C, Mylonas E, Penard-Lacronique V, Bischof O, Guillouf C | Yes |  |
| 34 | (Noh et al., 2017) | PTEN inhibits replicative senescence-induced MMP-1 expression by regulating NOX4-mediated ROS in human dermal fibroblasts | Noh EM, Kim JM, Hong OY, Song HK, Kim JS, Kwon KB, Lee YR | Yes |  |
| 35 | (Aarts et al., 2017) | Coupling shRNA screens with single-cell RNA-seq identifies a dual role for mTOR in reprogramming-induced senescence | Aarts M, Georgilis A, Beniazza M, Beolchi P, Banito A, Carroll T, Kulisic M, Kaemena DF, Dharmalingam G, Martin N, Reik W, Zuber J, Kaji K, Chandra T, Gil J | Yes |  |
| 36 | Cell Rep. 2017 Oct 3;21(1):1-9. doi: 10.1016/j.celrep.2017.09.026 | AMPK Maintains Cellular Metabolic Homeostasis through Regulation of Mitochondrial Reactive Oxygen Species | Rabinovitch RC, Samborska B, Faubert B, Ma EH, Gravel SP, Andrzejewski S, Raissi TC, Pause A, St-Pierre J, Jones RG | No | Only mouse qPCR data |
| 37 | Aging Cell. 2017 Oct;16(5):1043-1050. doi: 10.1111/acel.12632. Epub 2017 Jul 11 | Analysis of individual cells identifies cell-to-cell variability following induction of cellular senescence | Wiley CD, Flynn JM, Morrissey C, Lebofsky R, Shuga J, Dong X, Unger MA, Vijg J, Melov S, Campisi J | No | Single-cell qPCR only |
| 38 | (Y. Y. Kim et al., 2017) | Cooperation between p21 and Akt is required for p53-dependent cellular senescence | Kim YY, Jee HJ, Um JH, Kim YM, Bae SS, Yun J | Yes |  |
| 39 | (Park & Shin, 2017) | Metformin alleviates ageing cellular phenotypes in Hutchinson-Gilford progeria syndrome dermal fibroblasts | Park SK, Shin OS | Yes |  |
| 40 | (Markopoulos et al., 2017) | Senescence-associated microRNAs target cell cycle regulatory genes in normal human lung fibroblasts | Markopoulos GS, Roupakia E, Tokamani M, Vartholomatos G, Tzavaras T, Hatziapostolou M, Fackelmayer FO, Sandaltzopoulos R, Polytarchou C, Kolettas E | Yes |  |
| 41 | (Alessio et al., 2017) | Misidentified Human Gene Functions with Mouse Models: The Case of the Retinoblastoma GeneFamily in Senescence | Alessio N, Capasso S, Ferone A, Di Bernardo G, Cipollaro M, Casale F, Peluso G, Giordano A, Galderisi U | Yes |  |
| 42 | Biochem Biophys Res Commun. 2017 Sep 16;491(2):337-342. doi: 10.1016/j.bbrc.2017.07.099. Epub 2017 Jul 18 | MKK4 activates non-canonical NFκB signaling by promoting NFκB2-p100 processing | Kim JS, Kim EJ, Kim HS, Kurie JM, Ahn YH | No | No qPCR in human fibroblasts |
| 43 | Cell Rep. 2017 Sep 5;20(10):2468-2479. doi: 10.1016/j.celrep.2017.08.048 | The ULK3 Kinase Is Critical for Convergent Control of Cancer-Associated Fibroblast Activation by CSL and GLI | Goruppi S, Procopio MG, Jo S, Clocchiatti A, Neel V, Dotto GP | No | No qPCR in human fibroblasts |
| 44 | Chest. 2017 Sep;152(3):618-626. doi: 10.1016/j.chest.2017.03.020. Epub 2017 Mar 21 | Mitochondrial Dysfunction in Airway Disease | Prakash YS, Pabelick CM, Sieck GC | No | Review |
| 45 | FASEB J. 2017 Sep;31(9):3882-3893. doi: 10.1096/fj.201700014R. Epub 2017 May 17 | Progerin sequestration of PCNA promotes replication fork collapse and mislocalization of XPA in laminopathy-related progeroid syndromes | Hilton BA, Liu J, Cartwright BM, Liu Y, Breitman M, Wang Y, Jones R, Tang H, Rusinol A, Musich PR, Zou Y | No | No qPCR |
| 46 | (Menicacci et al., 2017) | Chronic Resveratrol Treatment Inhibits MRC5 Fibroblast SASP-Related Protumoral Effects on Melanoma Cells | Menicacci B, Laurenzana A, Chillà A, Margheri F, Peppicelli S, Tanganelli E, Fibbi G, Giovannelli L, Del Rosso M, Mocali A | Yes |  |
| 47 | (Moghadam, Mesbah-Ardakani, & Nasr-Esfahani, 2017) | Effects of Oleo Gum Resin of Ferula assa-foetida L. on Senescence in Human Dermal Fibroblasts: - Asafoetida reverses senescence in fibroblasts | Moghadam FH, Mesbah-Ardakani M, Nasr-Esfahani MH | Yes |  |
| 48 | (Butt et al., 2017) | Protective role of vitamin E preconditioning of human dermal fibroblasts against thermal stress in vitro | Butt H, Mehmood A, Ali M, Tasneem S, Anjum MS, Tarar MN, Khan SN, Riazuddin S | Yes |  |
| 49 | Nucleic Acids Res. 2017 Aug 21;45(14):8403-8410. doi: 10.1093/nar/gkx561 | BRD4 inhibitors block telomere elongation | Wang S, Pike AM, Lee SS, Strong MA, Connelly CJ, Greider CW | No | No human fibroblasts |
| 50 | Aging Cell. 2017 Aug;16(4):870-887. doi: 10.1111/acel.12621. Epub 2017 Jun 8 | Reprogramming progeria fibroblasts re-establishes a normal epigenetic landscape | Chen Z, Chang WY, Etheridge A, Strickfaden H, Jin Z, Palidwor G, Cho JH, Wang K, Kwon SY, Doré C, Raymond A, Hotta A, Ellis J, Kandel RA, Dilworth FJ, Perkins TJ, Hendzel MJ, Galas DJ, Stanford WL | No | No information about reference gene on the paper. Refers to other article. |
| 51 | Aging Cell. 2017 Aug;16(4):773-784. doi: 10.1111/acel.12606. Epub 2017 May 17 | Caveolin-1 deficiency induces premature senescence with mitochondrial dysfunction | Yu DM, Jung SH, An HT, Lee S, Hong J, Park JS, Lee H, Lee H, Bahn MS, Lee HC, Han NK, Ko J, Lee JS, Ko YG | No | No qPCR in human fibroblasts |
| 52 | (Gizard et al., 2005) | The PPARγ-SETD8 axis constitutes an epigenetic, p53-independent checkpoint on p21-mediated cellular senescence | Shih CT, Chang YF, Chen YT, Ma CP, Chen HW, Yang CC, Lu JC, Tsai YS, Chen HC, Tan BC | Yes |  |
| 53 | Aging Cell. 2017 Aug;16(4):726-737. doi: 10.1111/acel.12602. Epub 2017 May 10 | Enhanced NOLC1 promotes cell senescence and represses hepatocellular carcinoma cell proliferation by disturbing the organization of nucleolus | Yuan F, Zhang Y, Ma L, Cheng Q, Li G, Tong T | No | No qPCR in human fibroblasts |
| 54 | Anticancer Res. 2017 Aug;37(8):4311-4318 | Immortalized Cancer-associated Fibroblasts Promote Prostate Cancer Carcinogenesis, Proliferation and Invasion | Yu S, Jiang Y, Wan F, Wu J, Gao Z, Liu D | No | No access |
| 55 | Cancer Res. 2017 Aug 1;77(15):4014-4025. doi: 10.1158/0008-5472.CAN-16-2885. Epub 2017 Jun 15 | IGFBP7 Deletion Promotes Hepatocellular Carcinoma | Akiel M, Guo C, Li X, Rajasekaran D, Mendoza RG, Robertson CL, Jariwala N, Yuan F, Subler MA, Windle J, Garcia DK, Lai Z, Chen HH, Chen Y, Giashuddin S, Fisher PB, Wang XY, Sarkar D | No | No human fibroblasts |
| 56 | (Lee et al., 2017) | Protective effect of Arthrospira platensis extracts against ultraviolet B-induced cellular senescence through inhibition of DNA damage and matrix metalloproteinase-1 expression in human dermal fibroblasts | Lee JJ, Kim KB, Heo J, Cho DH, Kim HS, Han SH, Ahn KJ, An IS, An S, Bae S | Yes |  |
| 57 | (Yu et al., 2017) | Melatonin regulates PARP1 to control the senescence-associated secretory phenotype (SASP) in human fetal lung fibroblast cells | Yu S, Wang X, Geng P, Tang X, Xiang L, Lu X, Li J, Ruan Z, Chen J, Xie G, Wang Z, Ou J, Peng Y, Luo X, Zhang X, Dong Y, Pang X, Miao H, Chen H, Liang H | Yes |  |
| 58 | PLoS One. 2017 Jul 26;12(7):e0181530. doi: 10.1371/journal.pone.0181530. eCollection 2017 | Ionizing radiation response of primary normal human lens epithelial cells | Hamada N | No | No qPCR data |
| 59 | (P. Wang et al., 2017) | FOXQ1 regulates senescence-associated inflammation via activation of SIRT1 expression | Wang P, Lv C, Zhang T, Liu J, Yang J, Guan F, Hong T | Yes |  |
| 60 | Breast Cancer Res. 2017 Jul 3;19(1):78. doi: 10.1186/s13058-017-0871-0 | The footprint of the ageing stroma in older patients with breast cancer | Brouwers B, Fumagalli D, Brohee S, Hatse S, Govaere O, Floris G, Van den Eynde K, Bareche Y, Schöffski P, Smeets A, Neven P, Lambrechts D, Sotiriou C, Wildiers H | No | No qPCR in human fibroblasts |
| 61 | Oncogene. 2017 Jun 15;36(24):3464-3476. doi: 10.1038/onc.2016.496. Epub 2017 Jan 23 | The novel MKL target gene myoferlin modulates expansion and senescence of hepatocellular carcinoma | Hermanns C, Hampl V, Holzer K, Aigner A, Penkava J, Frank N, Martin DE, Maier KC, Waldburger N, Roessler S, Goppelt-Struebe M, Akrap I, Thavamani A, Singer S, Nordheim A, Gudermann T, Muehlich S | No | No qPCR in human fibroblasts |
| 62 | (Yang, Wang, Ren, Chen, & Chen, 2017) | cGAS is essential for cellular senescence | Yang H2, Wang H, Ren J, Chen Q, Chen ZJ | Yes |  |
| 63 | (Dillinger, Straub, & Nemeth, 2017) | Nucleolus association of chromosomal domains is largely maintained in cellular senescencedespite massive nuclear reorganisation | Dillinger S, Straub T, Németh A | Yes |  |
| 64 | (R. Wang et al., 2017) | Rapamycin inhibits the secretory phenotype of senescent cells by a Nrf2-independent mechanism | Wang R, Yu Z, Sunchu B, Shoaf J, Dang I, Zhao S, Caples K, Bradley L, Beaver LM, Ho E, Löhr CV, Perez VI | Yes |  |
| 65 | Cell Death Differ. 2017 Jun;24(6):1017-1028. doi: 10.1038/cdd.2017.48. Epub 2017 Mar 31 | Δ133p53 represses p53-inducible senescence genes and enhances the generation of humaninduced pluripotent stem cells | Horikawa I, Park KY, Isogaya K, Hiyoshi Y, Li H, Anami K, Robles AI, Mondal AM, Fujita K, Serrano M, Harris CC | No | No qPCR in senescent fibroblasts |
| 66 | Genomics Inform. 2017 Jun;15(2):56-64. doi: 10.5808/GI.2017.15.2.56. Epub 2017 Jun 15 | Analysis of Gene Expression in Human Dermal Fibroblasts Treated with Senescence-Modulating COX Inhibitors | Han JA, Kim JI | No | No qPCR data |
| 67 | Mech Ageing Dev. 2017 Jun;164:82-90. doi: 10.1016/j.mad.2017.05.001. Epub 2017 May 2 | Skin melanocytes and fibroblasts show different changes in choline metabolism during cellular senescence | Windler C, Gey C, Seeger K | No | No qPCR data |
| 68 | Sci Rep. 2017 May 30;7(1):2475. doi: 10.1038/s41598-017-02419-3 | Anti-Aging Potentials of Methylene Blue for Human Skin Longevity | Xiong ZM, O'Donovan M, Sun L, Choi JY, Ren M, Cao K | No | No qPCR in senescent fibroblasts |
| 69 | Oncotarget. 2017 May 16;8(20):33475-33486. doi: 10.18632/oncotarget.16509 | Microrna-217 modulates human skin fibroblast senescence by directly targeting DNA methyltransferase 1 | Wang B, Du R, Xiao X, Deng ZL, Jian D, Xie HF, Li J | No | Only qPCR for miRNA expression |
| 70 | (Contrepois et al., 2017) | Histone variant H2A.J accumulates in senescent cells and promotes inflammatory gene expression | Contrepois K, Coudereau C, Benayoun BA, Schuler N, Roux PF, Bischof O, Courbeyrette R, Carvalho C, Thuret JY, Ma Z, Derbois C, Nevers MC, Volland H, Redon CE, Bonner WM, Deleuze JF, Wiel C, Bernard D, Snyder MP, Rübe CE, Olaso R, Fenaille F, Mann C | Yes |  |
| 71 | Oncotarget. 2017 May 9;8(19):30908-30921. doi: 10.18632/oncotarget.15889 | Dependence of p53-deficient cells on the DHX9 DExH-box helicase | Lee T, Pelletier J | No | No qPCR in human fibroblasts |
| 72 | J Gerontol A Biol Sci Med Sci. 2017 May 1;72(5):632-639. doi: 10.1093/gerona/glw150 | UVB-Induced Senescence of Human Dermal Fibroblasts Involves Impairment of Proteasome and Enhanced Autophagic Activity | Cavinato M, Koziel R, Romani N, Weinmüllner R, Jenewein B, Hermann M, Dubrac S, Ratzinger G, Grillari J, Schmuth M, Jansen-Dürr P | No | Not enough information |
| 73 | (Panda et al., 2017) | Identification of senescence-associated circular RNAs (SAC-RNAs) reveals senescencesuppressor CircPVT1 | Panda AC, Grammatikakis I, Kim KM, De S, Martindale JL, Munk R, Yang X, Abdelmohsen K, Gorospe M | Yes |  |
| 74 | (Sasaki, Itakura, & Toyoda, 2017) | Sialylation regulates myofibroblast differentiation of human skin fibroblasts | Sasaki N, Itakura Y, Toyoda M | Yes |  |
| 75 | J Cell Sci. 2017 Apr 15;130(8):1413-1420. doi: 10.1242/jcs.196469. Epub 2017 Mar 6 | Proline dehydrogenase promotes senescence through the generation of reactive oxygen species | Nagano T, Nakashima A, Onishi K, Kawai K, Awai Y, Kinugasa M, Iwasaki T, Kikkawa U, Kamada S | No | No qPCR data |
| 76 | (Latella et al., 2017) | DNA damage signaling mediates the functional antagonism between replicative senescence and terminal muscle differentiation | Latella L, Dall'Agnese A, Boscolo FS, Nardoni C, Cosentino M, Lahm A, Sacco A, Puri PL | Yes |  |
| 77 | BMC Syst Biol. 2017 Mar 15;11(1):36. doi: 10.1186/s12918-017-0417-1 | Systematic identification of an integrative network module during senescence from time-series gene expression | Park C, Yun SJ, Ryu SJ, Lee S, Lee YS, Yoon Y, Park SC | No | No qPCR data |
| 78 | (Xie et al., 2017) | miR-377 induces senescence in human skin fibroblasts by targeting DNA methyltransferase 1 | Xie HF, Liu YZ, Du R, Wang B, Chen MT, Zhang YY, Deng ZL, Li J | Yes (see comments) | Different gene for the miRNA and a protein-coding gene. Only included GAPDH (for protein-coding) and not U6 (for miRNA) |
| 79 | (Jung et al., 2017) | The Ubiquitin-like with PHD and Ring Finger Domains 1 (UHRF1)/DNA Methyltransferase 1 (DNMT1) Axis Is a Primary Regulator of Cell Senescence | Jung HJ, Byun HO, Jee BA, Min S, Jeoun UW, Lee YK, Seo Y, Woo HG, Yoon G | Yes |  |
| 80 | Arch Dermatol Res. 2017 Mar;309(2):123-132. doi: 10.1007/s00403-016-1713-0. Epub 2017 Jan 11 | Senescence in the lesional fibroblasts of non-segmental vitiligo patients | Rani S, Bhardwaj S, Srivastava N, Sharma VL, Parsad D, Kumar R | No | No qPCR in senescent fibroblasts |
| 81 | (Al-Khalaf & Aboussekhra, 2017) | p16INK4A induces senescence and inhibits EMT through microRNA-141/microRNA-146b-5p-dependent repression of AUF1 | Al-Khalaf HH, Aboussekhra A | Yes |  |
| 82 | (Youn, Kim, Han, An, & Ahn, 2017) | 23-Hydroxytormentic acid protects human dermal fibroblasts by attenuating UVA-induced oxidative stress | Youn HJ, Kim KB, Han HS, An IS, Ahn KJ | Yes |  |
| 83 | PLoS One. 2017 Feb 3;12(2):e0171431. doi: 10.1371/journal.pone.0171431. eCollection 2017 | Potential roles of DNA methylation in the initiation and establishment of replicative senescencerevealed by array-based methylome and transcriptome analyses | Sakaki M, Ebihara Y, Okamura K, Nakabayashi K, Igarashi A, Matsumoto K, Hata K, Kobayashi Y, Maehara K | No | Not enough information |
| 84 | (Cui et al., 2017) | miR-34a Inhibits Lung Fibrosis by Inducing Lung Fibroblast Senescence | Cui H, Ge J, Xie N, Banerjee S, Zhou Y, Antony VB, Thannickal VJ, Liu G | Yes |  |
| 85 | Biogerontology. 2017 Feb;18(1):69-84. doi: 10.1007/s10522-016-9662-8. Epub 2016 Oct 18 | Telomere length is regulated by FGF-2 in human embryonic stem cells and affects the life span of its differentiated progenies | Zou Y, Tong HJ, Li M, Tan KS, Cao T | No | No qPCR in senescent fibroblasts |
| 86 | Biogerontology. 2017 Feb;18(1):55-68. doi: 10.1007/s10522-016-9661-9. Epub 2016 Oct 12 | Increase in tumor suppressor Arf compensates gene dysregulation in in vitro aged adipocytes | Hasan AU, Ohmori K, Hashimoto T, Kamitori K, Yamaguchi F, Konishi K, Noma T, Igarashi J, Yamashita T, Hirano K, Tokuda M, Minamino T, Nishiyama A, Kohno M | No | No qPCR in senescent fibroblasts |
| 87 | (Zainuddin, Chua, Tan, Jaafar, & Makpol, 2017) | γ-Tocotrienol prevents cell cycle arrest in aged human fibroblast cells through p16INK4a pathway | Zainuddin A, Chua KH, Tan JK, Jaafar F, Makpol S | Yes |  |
| 88 | PLoS One. 2017 Jan 26;12(1):e0169189. doi: 10.1371/journal.pone.0169189. eCollection 2017 | A Novel Lamin A Mutant Responsible for Congenital Muscular Dystrophy Causes Distinct Abnormalities of the Cell Nucleus | Barateau A, Vadrot N, Vicart P, Ferreiro A, Mayer M, Héron D, Vigouroux C, Buendia B | No | No qPCR data |
| 89 | Biochem J. 2017 Jan 15;474(2):281-300. doi: 10.1042/BCJ20160459. Epub 2016 Oct 19 | Loss of lamin B receptor is necessary to induce cellular senescence | Lukášová E, Kovarˇík A, Bacˇíková A, Falk M, Kozubek S | No | No access |
| 90 | Oncogene. 2017 Jan 12;36(2):219-230. doi: 10.1038/onc.2016.193. Epub 2016 May 30 | Foxp3 is a key downstream regulator of p53-mediated cellular senescence | Kim JE, Shin JS, Moon JH, Hong SW, Jung DJ, Kim JH, Hwang IY, Shin YJ, Gong EY, Lee DH, Kim SM, Lee EY, Kim YS, Kim D, Hur D, Kim TW, Kim KP, Jin DH, Lee WJ | No | No human fibroblasts |
| 91 | Adv Exp Med Biol. 2017;967:1-11. doi: 10.1007/978-3-319-63245-2_1 | Adventitial Fibroblast Nox4 Expression and ROS Signaling in Pulmonary Arterial Hypertension | Barman SA, Fulton D | No | Review |
| 92 | (Marmisolle et al., 2017) | Reciprocal regulation of acetyl-CoA carboxylase 1 and senescence in human fibroblasts involves oxidant mediated p38 MAPK activation | Marmisolle I, Martínez J, Liu J, Mastrogiovanni M, Fergusson MM, Rovira II, Castro L, Trostchansky A, Moreno M, Cao L, Finkel T, Quijano C | Yes |  |
| 93 | Biochim Biophys Acta. 2017 Jan;1864(1):177-190. doi: 10.1016/j.bbamcr.2016.11.008. Epub 2016 Nov 11 | ATM-ROS-iNOS axis regulates nitric oxide mediated cellular senescence | Bagheri M, Nair RR, Singh KK, Saini DK | No | No qPCR in senescent fibroblasts |
| 94 | (Durani et al., 2017) | Piper betle L. Modulates Senescence-Associated Genes Expression in Replicative Senescent Human Diploid Fibroblasts | Durani LW, Khor SC, Tan JK, Chua KH, Mohd Yusof YA, Makpol S | Yes |  |
| 95 | Curr Protein Pept Sci. 2017;18(12):1224-1231. doi: 10.2174/1389203717666160915162238 | Cell Cycle Arrest as a Therapeutic Target of Acute Kidney Injury | Wang WG, Sun WX, Gao BS, Lian X, Zhou HL | No | Review |
| 96 | Mech Ageing Dev. 2017 Jan;161(Pt A):51-65. doi: 10.1016/j.mad.2016.06.009. Epub 2016 Jun 21 | 8-Oxoguanine DNA glycosylase1-driven DNA repair-A paradoxical role in lung aging | German P, Saenz D, Szaniszlo P, Aguilera-Aguirre L, Pan L, Hegde ML, Bacsi A, Hajas G, Radak Z, Ba X, Mitra S, Papaconstantinou J, Boldogh I | No | No qPCR data |
| 97 | Methods Mol Biol. 2017;1534:165-173 | Detection of Nucleotide Disbalance in Cells Undergoing Oncogene-Induced Senescence | Nikiforov MA, Shewach DS | No | No access |
| 98 | Methods Mol Biol. 2017;1534:127-137 | Detection of the Ubiquitinome in Cells Undergoing Oncogene-Induced Senescence | Zhu H, Le L, Tang HY, Speicher DW, Zhang R | No | No access |
| 99 | Methods Mol Biol. 2017;1534:99-109 | Detecting the Senescence-Associated Secretory Phenotype (SASP) by High Content Microscopy Analysis | Hari P, Acosta JC | No | No access |
| 100 | Methods Mol Biol. 2017;1534:89-98 | Autophagy Detection During Oncogene-Induced Senescence Using Fluorescence Microscopy | Narita M, Narita M | No | No access |
| 101 | Methods Mol Biol. 2017;1534:69-78 | Detection of Dysfunctional Telomeres in Oncogene-Induced Senescence | Patel PL, Herbig U | No | No access |
| 102 | Methods Mol Biol. 2017;1534:17-30 | Senescence Phenotypes Induced by Ras in Primary Cells | Lau L, David G | No | No access |
| 103 | (T. Wang et al., 2017) | Senescent Carcinoma-Associated Fibroblasts Upregulate IL8 to Enhance Prometastatic Phenotypes | Wang T, Notta F, Navab R, Joseph J, Ibrahimov E, Xu J, Zhu CQ, Borgida A, Gallinger S, Tsao MS | Yes |  |
| 104 | (Xia, Sun, Xie, & Shu, 2017) | mTOR Inhibition Rejuvenates the Aging Gingival Fibroblasts through Alleviating Oxidative Stress | Xia Y, Sun M, Xie Y, Shu R | Yes |  |
| 105 | (Mistriotis et al., 2017) | NANOG Reverses the Myogenic Differentiation Potential of Senescent Stem Cells by Restoring ACTIN Filamentous Organization and SRF-Dependent Gene Expression | Mistriotis P, Bajpai VK, Wang X, Rong N, Shahini A, Asmani M, Liang MS, Wang J, Lei P, Liu S, Zhao R, Andreadis ST | Yes |  |

- **Table S2: RNAseq datasets used for the Selection of New and Stable Reference Genes**

| Reference | Accession Number | Type of Senescence | Replicates | Strain | Senescence testing | Remarks |
| --- | --- | --- | --- | --- | --- | --- |
| (Abdelmohsen et al., 2013) | GSE77675 | Replicative | 1 | WI-38 | bgal (not quantified), size and shape, western blot of p21, p53, Sirt1 and HuR |  |
| (Alspach et al., 2014) | GSE56293G | Replicative | 3 | BJ | SA-bgal (>60%) | Only subset of samples used (young versus old) |
| (Capell et al., 2016) | GSE78138 | OIS | 1 | IMR90 | SA-bgal (>85%) and expression of CDKN2A, CDK2 and LMNB1 |  |
| (Dikovskaya et al., 2015) | GSE70668 | OIS | 3 | IMR90 | No markers measure at time of harvest for RNA-seq (day 4) but at day 10 | Synchronized cells |
| (Duarte et al., 2014) | GSE55949 | OIS | 1 | IMR90 | SA-bgal (not quantified). EdU incorporation and SAHFs. |  |
| (Hernandez-Segura et al., 2017) | E-MTAB-5403 | IRIS | 6 | HCA-2 | SA-bgal (>75%) and EdU incorporation (<20%) | Dataset included day 4, day 10, day 20 post-irradiation and quiescence. |
| (Herranz et al., 2015) | GSE61130 | OIS | 3 | IMR90 | SA-bgal (>55%), EdU incorporation (<20%) and expression of p16 and p21 | Only subset of samples used (young versus old with no genetic manipulation) |
| (Marthandan et al., 2016) | GSE63577 | Replicative | 3 | BJ, WI-38, IMR90, MRC-5, HFF | SA-bgal (MRC-5 >75%; HFF >80%; all the other strains not specified), growth curve | Only subset of samples used (proliferating and last timepoint). |
| (Marthandan et al., 2015) | GSE64553 | Replicative | 3 | MRC-5, HFF | SA-bgal (HFF >60%, MRC5 ~20%), growth curve | Only HFF included |
| (Rai et al., 2014) | GSE53356 | Replicative | 2 | IMR90 | SA-bgal (~50%), PML and cyclin A staining | Only subset of samples used (proliferating and senescence) |

- **Table S3: Reference Gene Candidates for Senescence Experiments in Human Fibroblasts as calculated by RNAseq (p-value Shapiro-Wilk test >=0.6 and coefficient of variation <=20)**

| **Gene** | **ENSEMBL ID** | **p-value Shapiro-Wilk** | **Coeff. Var.** |
| --- | --- | --- | --- |
| TMEM199 | ENSG00000244045 | 0.992104732 | 13.97273811 |
| WDR55 | ENSG00000120314 | 0.965647995 | 14.54151374 |
| RBCK1 | ENSG00000125826 | 0.962533746 | 18.86296949 |
| L3MBTL2 | ENSG00000100395 | 0.956988816 | 14.26635023 |
| VAMP7 | ENSG00000124333 | 0.956925687 | 14.00947654 |
| ZNF621 | ENSG00000172888 | 0.954363932 | 16.15136195 |
| SMUG1 | ENSG00000123415 | 0.937249679 | 16.38906442 |
| ANKS3 | ENSG00000168096 | 0.9118185 | 15.14601966 |
| ANXA7 | ENSG00000138279 | 0.902876382 | 16.38624072 |
| ATF1 | ENSG00000123268 | 0.888430908 | 19.68042036 |
| GOT2 | ENSG00000125166 | 0.87877521 | 19.25107318 |
| CSNK1G3 | ENSG00000151292 | 0.877838971 | 18.78984367 |
| EIF4E2 | ENSG00000135930 | 0.874171222 | 12.06728814 |
| FAN1 | ENSG00000198690 | 0.871537163 | 19.19644188 |
| KDM4A | ENSG00000066135 | 0.864697228 | 14.60026102 |
| COPS5 | ENSG00000121022 | 0.855040408 | 12.55867679 |
| ZNF740 | ENSG00000139651 | 0.852317802 | 18.13795305 |
| ARL14EP | ENSG00000152219 | 0.843095954 | 18.67398053 |
| AIP | ENSG00000110711 | 0.842320989 | 17.198364 |
| CBR4 | ENSG00000145439 | 0.823553493 | 19.84217305 |
| ZNF720 | ENSG00000197302 | 0.823438108 | 17.6243384 |
| KLHDC8B | ENSG00000185909 | 0.812874236 | 19.15397685 |
| SLC25A16 | ENSG00000122912 | 0.797624647 | 13.26182813 |
| TIMM22 | ENSG00000177370 | 0.776107364 | 12.52514539 |
| PRMT9 | ENSG00000164169 | 0.772024116 | 15.70856333 |
| DPH2 | ENSG00000132768 | 0.76875196 | 19.7137143 |
| LDOC1L | ENSG00000188636 | 0.745931373 | 19.3809777 |
| PSMD5 | ENSG00000095261 | 0.741904705 | 12.16720477 |
| SMIM12 | ENSG00000163866 | 0.736045105 | 10.85502721 |
| RSPRY1 | ENSG00000159579 | 0.735276846 | 11.78324636 |
| ZMAT5 | ENSG00000100319 | 0.732576166 | 13.99749692 |
| WDR59 | ENSG00000103091 | 0.720164699 | 18.73933865 |
| PRPF18 | ENSG00000165630 | 0.717469799 | 16.43208533 |
| ASPSCR1 | ENSG00000169696 | 0.71312373 | 19.0486751 |
| C1orf52 | ENSG00000162642 | 0.704561897 | 18.64914126 |
| TMEM258 | ENSG00000134825 | 0.703016258 | 19.51305175 |
| AZI2 | ENSG00000163512 | 0.694411501 | 18.34750224 |
| EIF2D | ENSG00000143486 | 0.693957848 | 19.08476182 |
| OTUD5 | ENSG00000068308 | 0.693854725 | 18.56090617 |
| CAAP1 | ENSG00000120159 | 0.690991545 | 17.87405802 |
| BAG5 | ENSG00000166170 | 0.690290665 | 16.99334938 |
| DDX59 | ENSG00000118197 | 0.688542721 | 17.71357018 |
| RMND5B | ENSG00000145916 | 0.688062507 | 19.34643328 |
| AIMP1 | ENSG00000164022 | 0.685937381 | 19.64689963 |
| FAM200A | ENSG00000221909 | 0.683947263 | 14.78323172 |
| CNDP2 | ENSG00000133313 | 0.677122518 | 17.05549567 |
| RBM48 | ENSG00000127993 | 0.675431206 | 18.19036701 |
| SEC22A | ENSG00000121542 | 0.671178011 | 16.16242902 |
| RAB34 | ENSG00000109113 | 0.665984878 | 13.05383819 |
| RPP14 | ENSG00000163684 | 0.661912187 | 13.30072537 |
| STX6 | ENSG00000135823 | 0.660327669 | 18.57101371 |
| NDEL1 | ENSG00000166579 | 0.657561531 | 13.38361634 |
| WDR82 | ENSG00000164091 | 0.639933236 | 16.10688471 |
| TAF6L | ENSG00000162227 | 0.638825688 | 16.08824899 |
| MTHFSD | ENSG00000103248 | 0.632147877 | 12.85677229 |
| GFM1 | ENSG00000168827 | 0.631478673 | 11.66401232 |
| RXRB | ENSG00000204231 | 0.629197108 | 15.36507242 |
| TBRG1 | ENSG00000154144 | 0.627035871 | 19.45280085 |
| MZT2B | ENSG00000152082 | 0.618335304 | 19.94142392 |
| ARL1 | ENSG00000120805 | 0.617259292 | 17.48037976 |
| PRKAB1 | ENSG00000111725 | 0.608729474 | 13.8119742 |
| WDR81 | ENSG00000167716 | 0.604819613 | 19.23757424 |
| NGLY1 | ENSG00000151092 | 0.603031906 | 14.32387196 |
| WWP1 | ENSG00000123124 | 0.602813917 | 17.0637983 |
| DPH7 | ENSG00000148399 | 0.600901629 | 16.75430269 |

- **Table S4: Samples used for qPCR Experiments**

| **Experiment** | **Cell Type** | **Samples** | **Replicates** |
| --- | --- | --- | --- |
| 1 | BJ | Control (prolif.) | 3 |
| 1 | BJ | IR (day 10) | 3 |
| 2 | BJ | Control (prolif.) | 3 |
| 2 | BJ | Doxorubicin | 3 |
| 3 | BJ | Control (prolif.) | 3 |
| 3 | BJ | Doxorubicin | 3 |
| 4 | BJ | Control (prolif.) | 3 |
| 4 | BJ | 5-aza-deoxycytidine | 3 |
| 4 | BJ | Replicative Senescence (PD55) | 3 |
| 5 | HCA2 | Control (prolif.) | 3 |
| 5 | HCA2 | IR (day 4) | 3 |
| 5 | HCA2 | IR (day 10) | 3 |
| 5 | HCA2 | Doxorubicin | 3 |
| 6 | HCA2 | Control (prolif.) | 3 |
| 6 | HCA2 | IR (day 4) | 3 |
| 6 | HCA2 | IR (day 10) | 3 |
| 6 | HCA2 | IR (day 20) | 3 |
| 6 | HCA2 | Quiescence | 3 |
| 7 | IMR90 | Control (prolif.) | 3 |
| 7 | IMR90 | IR (day 4) | 3 |
| 7 | IMR90 | IR (day 10) | 3 |
| 7 | IMR90 | Doxorubicin | 3 |
| 8 | IMR90 | Control (prolif.) | 3 |
| 8 | IMR90 | Doxorubicin | 3 |
| 9 | WI38 | Control (vehicle) | 3 |
| 9 | WI38 | HDAC3 inhibitor (RGFP966) | 3 |
| 9 | WI38 | Doxorubicin | 3 |
| 10 | WI38 | Control (vehicle) | 3 |
| 10 | WI38 | HDAC 1 and 3 inhibitor (entinostat) | 3 |
| 10 | WI38 | HDAC3 inhibitor (RGFP966) | 3 |
| 10 | WI38 | SAHA | 3 |
| 11 | WI38 | Control (prolif.) | 2 |
| 11 | WI38 | IR (day 10) | 2 |
| 11 | WI38 | Doxorubicin | 2 |

- **Table S5: Gene Functions of Reference Gene Candidates and Assays used to Measure Their Expression**

| **Gene Name (ENSEMBL ID)** | **Name** | **Function** | **Localization** | **Forward** | **Reverse** | **Amplicon size (bp)** | **Variants targeted** |
| --- | --- | --- | --- | --- | --- | --- | --- |
| ACTB (ENSG00000075624) | Actin Beta | Cell motility, structure integrity and intercellular signaling | Chromosome 7: 5,527,147-5,563,784 reverse strand | ccaaccgcgagaagatga | ccagaggcgtacagggatag | 97 | 6 out of 19 |
| GAPDH (ENSG00000111640) | Glyceraldehyde-3-Phosphate Dehydrogenase | Glucose metabolism | Chromosome 12: 6,533,927-6,538,374 forward strand | agccacatcgctcagacac | gcccaatacgaccaaatcc | 66 | 7 out of 11 |
| RPLP0 (ENSG00000089157) | Ribosomal Protein Lateral Stalk Subunit P0 | Protein synthesis. Component of ribosome (60S subunit) | Chromosome 12: 120,196,686-120,201,235 reverse strand | tctacaaccctgaagtgcttgat | caatctgcagacagacactgg | 96 | 12 out of 27 |
| TUBA1A (ENSG00000167552) | Tubulin Alpha 1a | Cytoeskeletal protein involved in cell cycle and mitosis | Chromosome 12: 49,184,796-49,189,324 reverse strand | cttcgtctccgccatcag | cgtgttccaggcagtagagc | 127 and 215 | 4 out of 9 |
| VCL (ENSG00000035403) | Vinculin | Cytoeskeletal protein associated with cell-cell and cell-matrix junctions | Chromosome 10: 73,995,193-74,121,363 forward strand | gatgaagctcgcaaatggtc | tctgcctcagctacaacacct | 77 | 3 out of 7 |
| L3MBTL2 (ENSG00000100395) | L(3)Mbt-Like Protein 2, Polycomb Repressive Complex 1 Subunit | Putative Polycomb group protein, involved in transcriptional repression | Chromosome 22: 41,205,205-41,231,271 forward strand | ccaagaccaagaggttctgc | tttggtcggtggttttcc | 104 | 7 out of 9 |
| RBCK1 (ENSG00000125826) | RANBP2-Type And C3HC4-Type Zinc Finger Containing 1 | E3-ubiquitin-protein ligase, promoting ubiquitination and degradation of its targets by the proteasome | Chromosome 20: 407,498-430,966 forward strand | agtgtctgcacaccttctgc | gcacgagtaggtgttgtcaatg | 101 | 2 out of 15 |
| TMEM199 (ENSG00000244045) | Transmembrane Protein 199 | May be involved in ER-Golgi homeostasis | Chromosome 17: 28,357,581-28,363,683 forward strand | caccagcatctgagagaaagg | ccgtggaggcttcacaac | 96 | 5 out of 9 |
| VAMP7 (ENSG00000124333) | Vesicle Associated Membrane Protein 7 | Targeting and fusion of target membranes to lysosomes | Chromosome X: 155,881,293-155,943,769 forward strand | caaacatgcttggtgtggag | aaattaaaggctcgggaacg | 63 | 5 out of 5 |
| WDR55 (ENSG00000120314) | WD Repeat Domain 55 | Nucleolar protein that modulates rRNA syntesis | Chromosome 5: 140,664,676-140,674,124 forward strand | ggaagacatcgtgctggaag | tggcaagagtaggaaaagacg | 114 | 2 out of 5 |

*Data taken from ENSEMBL ([www.ensembl.org](http://www.ensembl.org)) and GeneCards ([www.genecards.org](http://www.genecards.org)) on August 20^th^ 2018.

- **Table S6: Slope, r2 and PCR efficiency calculated for each primer set used**

| **Gene Target** | **Slope** | **r^2^** | **Efficiency** |
| --- | --- | --- | --- |
| TUBA1A | -3.4521 | 0.9997 | 95 |
| ACTB | -3.3676 | 0.9988 | 98 |
| VCL | -3.3732 | 0.9994 | 98 |
| RPLP0 | -3.3822 | 0.9996 | 98 |
| WDR55 | -3.4362 | 0.9985 | 95 |
| VAMP7 | -3.3977 | 0.9983 | 97 |
| RBCK1 | -3.6752 | 0.9788 | 87 |
| TMEM199 | -3.387 | 0.9959 | 97 |
| L3MBTL2 | -3.4345 | 0.9959 | 96 |
| GAPDH | -3.4428 | 0.9994 | 95 |

**SUPPLEMENTARY FIGURES**


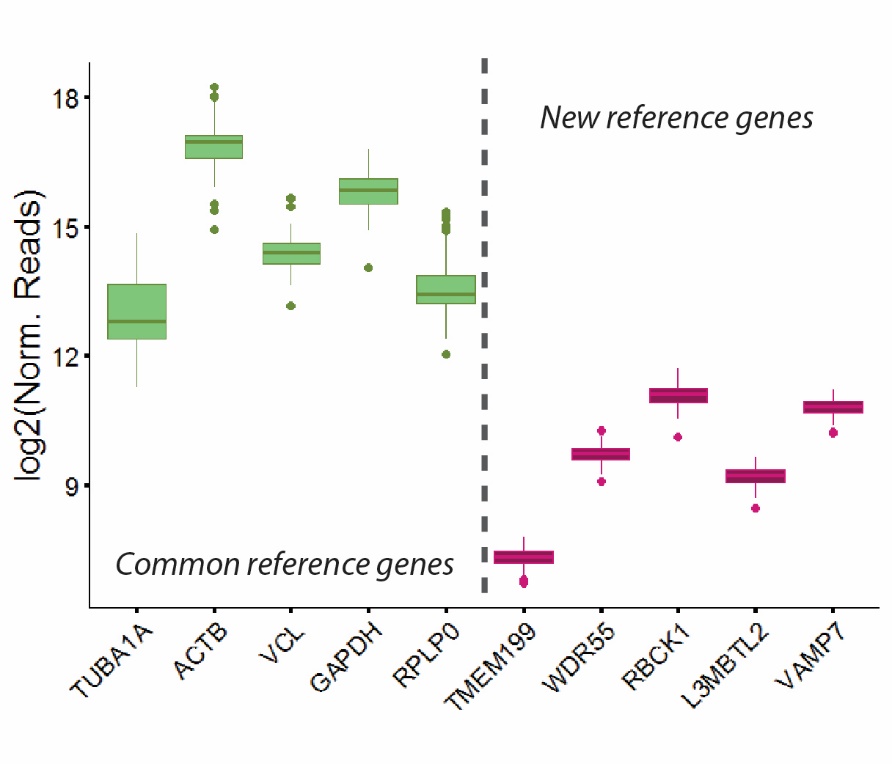


**Figure S1: Average expression of common and new reference gene candidates.** The logarithm of the normalized expression (normalized read counts using size factors as calculated in (Love et al., 2014)) in the five commonly used (green) and the five newly proposed (dark pink) reference genes is shown here.


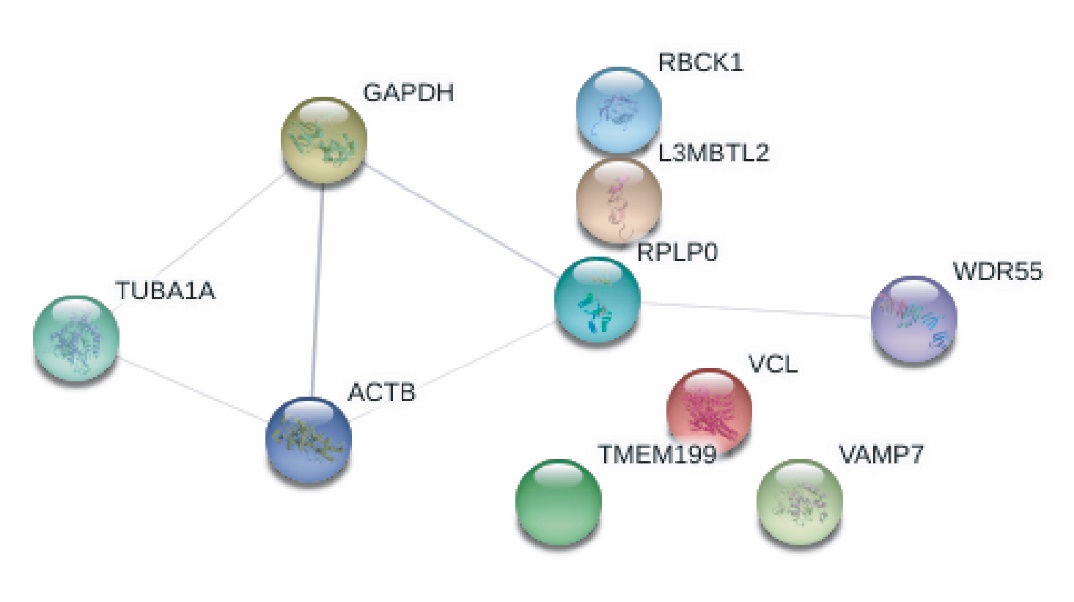


**Figure S2.** **Co-regulation of Reference Gene Candidates.** The co-regulation of the ten reference gene candidates was inquired using the online software STRING-db. Each node represents a protein and the connectors represent protein-protein associations (i.e. proteins contributing to shared function and not necessarily physically binding). The thickness of the connector represents the confidence of the association: low (0.150), medium (0.400), high (0.700) and highest (0.900). Only the association between ACTB and GAPDH was medium, all the others showed low confidence. Downloaded from <https://string-db.org/> on 30/08/2018.


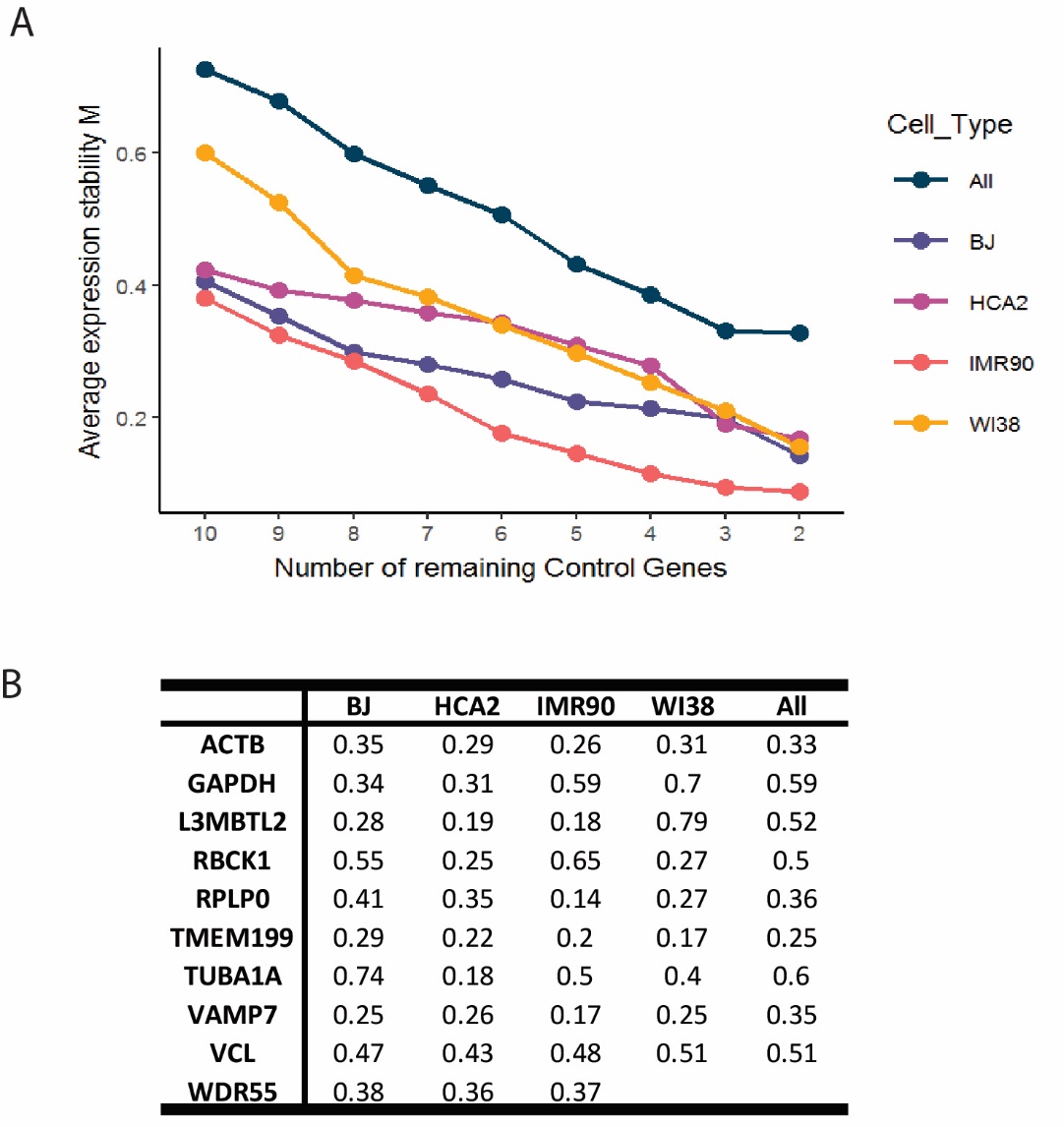


**Figure S3: M-values and Stability values as calculated by geNorm and NormFinder, respectively.** The results of the analysis for each reference gene candidate tested for every fibroblast strain. A. Average expression stability (M-value) after the stepwise exclusion of the least stable reference gene candidate for each fibroblast strain. B. Stability values for each reference gene candidate studied as calculated by NormFinder.
